# High folate receptor expression in gliomas can be detected *in vivo* using folate-based positron emission tomography with high tumor-to-brain uptake ratio divulging potential future targeting possibilities

**DOI:** 10.3389/fimmu.2023.1145473

**Published:** 2023-05-18

**Authors:** Maxwell W. G. Miner, Heidi Liljenbäck, Jenni Virta, Salli Kärnä, Riikka Viitanen, Petri Elo, Maria Gardberg, Jarmo Teuho, Piritta Saipa, Johan Rajander, Hasan Mansour A Mansour, Nathan A. Cleveland, Philip S. Low, Xiang-Guo Li, Anne Roivainen

**Affiliations:** ^1^ Turku PET Centre, University of Turku, Turku, Finland; ^2^ Turku Center for Disease Modeling, University of Turku, Turku, Finland; ^3^ Department of Pathology, Turku University Hospital and Institute of Biomedicine, University of Turku, Turku, Finland; ^4^ Turku PET Centre, Turku University Hospital, Turku, Finland; ^5^ Department of Medical Physics, Turku University Hospital, Turku, Finland; ^6^ Accelerator Laboratory, Turku PET Centre, Åbo Akademi University, Turku, Finland; ^7^ Department of Chemistry, Purdue University, West Lafayette, IN, United States; ^8^ Department of Chemistry, University of Turku, Turku, Finland; ^9^ InFLAMES Research Flagship Center, University of Turku, Turku, Finland

**Keywords:** fluorine-18, folate receptor, glioma, PET, ^18^F-labelled folate, brain tumor

## Abstract

**Introduction:**

Non-invasive imaging techniques such as positron emission tomography (PET) are extremely important for cancer detection and characterization especially for difficult to biopsy or extremely delicate organs such as the brain. The folate analogue 1,4,7-triazacylononane-1,4,7-triacetic acid-conjugated folate radiolabeled with aluminum fluoride-18 ([^18^F]FOL) has been previously shown to accumulate preferentially in tumor cells with an overexpression of folate receptors (FRs) and here was investigated for its ability to detect orthotopic gliomas in a rat model. In addition, we studied the expression of FRs in human glioblastoma samples to investigate if an analogous relationship may exist.

**Methods:**

Nine BDIX rats were injected with BT4C rat glioma cells into the right hemisphere of the brain. Animals were imaged with gadolinium-enhanced magnetic resonance imaging at on days prior to PET/computed tomography (CT) imaging. Animals were divided into two groups, and were PET/CT imaged with either [^18^F]FOL or 2-deoxy-2-18F-fluoro-D-glucose ([^18^F]FDG) on 19 and 32-days post glioma grafting. Two subjects were also PET/CT imaged with [^18^F]FOL on day 16. Biodistribution was studied and brains were cryosectioned for autoradiography, immunofluorescence, and histological studies. Patient-derived paraffin-embedded glioblastomas were sectioned and stained with similar methods.

**Results:**

PET imaging showed an increase of [18F]FOL tumor-to-brain uptake ratio (TBR) over the study duration from day 16/19 (3.3 ± 0.9) increasing to 5.7 ± 1.0 by day 32. [^18^F]FDG PET-imaged rats had a consistent TBR of 1.6 ± 0.1 throughout the study. Ex vivo autoradiography results revealed an exceptionally high TBR of 116.1 ± 26.9 for [^18^F]FOL while the [18F]FDG values were significantly lower giving 2.9 ± 0.6 (P<0.0001). Immunostaining demonstrated an increased presence of FR-α in the BT4C gliomas versus the contralateral brain tissue, while FR-β was present only on glioma periphery. Human sections assayed showed similar FRs expression characteristics.

**Conclusion:**

This study shows upregulation of FR-α inside glioma regions in both human and animal tissue, providing a biochemical basis for the observed increased [^18^F]FOL uptake in animal PET images. These results suggest that FRs targeting imaging and therapeutic compounds may possess clinically relevant translational abilities for the detection and treatment of gliomas.

## Graphical abstract

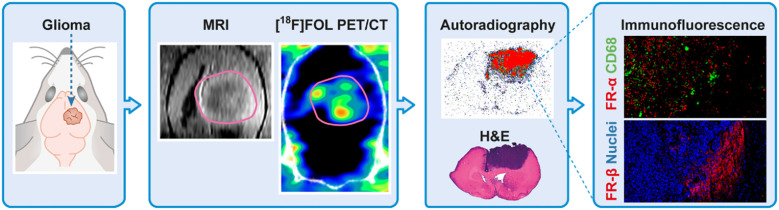

## 1 Introduction

Folate intake *via* dietary or supplemental means is essential for human health due to its requirement in the synthesis of nucleotide bases and some amino acids (1). Folate uptake is mediated in almost all cells by a reduced folate carrier (2), but in a few cells this uptake is augmented by a much higher affinity folate receptors (FRs) that transport the vitamin across the cell membrane *via* receptor-mediated endocytosis (3). Due to its requirement for DNA synthesis, it comes as no surprise that many different aggressive cancer cell lines have an overexpression of these receptors to assist with an increased rate of folate consumption (4). The relationship between FRs and cancer has been widely investigated for its potential in imaging and FR-targeted oncology therapies (5–7). Folate receptor alpha (FR-α) in particular has been the focus of much of this research and many studies have found an association between poor patient prognosis and high levels of FR-α expression (8, 9). Another important and common FR, the folate receptor beta (FR-β) is expressed highly on activated macrophage populations, which accumulate at inflamed sites (10). Pro-inflammatory signaling is common not only to many different diseases, but often also a result of rapidly dividing out of control cells (11). Identifying cancers with an overexpression of FRs in a non-invasive way, such as with positron emission tomography/computed tomography (PET/CT) imaging, can be useful for discerning whether a FR-targeted therapy may be appropriate for the patient. For particularly delicate organs, such as the brain, classifying biochemical traits without invasive biopsies that require tissue removal can be a much-welcomed alternative for investigative examination. A useful approach to radiopharmaceutical design is too often radiolabel naturally occurring metabolites (or analogue thereof) or radiolabel ligands that can bind to over-expressed receptors on the target tissue.

Gliomas are a group of brain tumors originating from the glial cells whose normal responsibilities include providing nutrients to neurons, forming general brain structure, insulation, and detection of dead neurons or pathogens (12). They make up a large majority of all brain tumors (13) and are classified based on histological and molecular characteristics. The diffuse gliomas are characterized by tumor cell infiltration into the surrounding brain and the most common diffuse glioma diagnosis among adults is the highly malignant glioblastoma (14). Being particularly suited to absorbing and providing nutrient transfer to neurons, when the division of glial cells becomes uncontrolled and cancerous they already have access to the tools required to sustain proliferative growth. When positron emission tomography (PET) imaging these tumors, however, it is particularly important to detect and distinguish tumor boundaries from healthy tissue to avoid missing small tumor masses in multifocal gliomas and lower the probability of early recurrence. This can be accomplished by selecting a PET imaging radiopharmaceutical that has high uptake in cancer cells and low uptake in other cells, but no radiopharmaceutical is perfect. The scientific community is constantly testing small modifications to existing radiopharmaceuticals as well as entirely novel compounds to improve PET imaging capabilities.

Glucose uptake in the brain under normal conditions is generally quite high, which is a double-edged sword for detecting small abnormalities (*e.g.* a new small lesion) in glucose consumption with glucose analogue 2-deoxy-2-[^18^F]fluoro-D-glucose ([^18^F]FDG). On one side, the high uptake of the brain allows for a large influx of the radiopharmaceutical making imaging the organ comparatively easy. On the other side, a high background signal due to the basal uptake of [^18^F]FDG in healthy tissue can make detecting small areas with increases in uptake not as obvious. Delineation between tumor tissue and healthy tissue is an important aspect of PET imaging cancer. Tumor-to-background or, in this case, tumor-to-brain ratio (TBR) can be used to quantify PET imaging radiopharmaceutical contrast. Furthermore, being able to more accurately map the contours of the tumor or detect secondary smaller malignant growths can be beneficial to patient outcomes. It can aid with treatment planning, surgery, monitor the success of surgery, and even lower the chance of additional relapses stemming from overlooked tumor tissue. In this study, we investigated the folate analogue PET imaging radiopharmaceutical aluminum fluoride-18-labelled 1,4,7-triazacylononane-1,4,7-triacetic acid-conjugated folate ([^18^F]FOL) for its ability to detect gliomas in a BT4C-rat-glioma model, where tumor histology and behavior highly resembles that found in human glioblastoma. By first using contrast-enhanced magnetic resonance imaging (MRI) to accurately delineate tumor boundaries prior to PET imaging with either [^18^F]FOL or [^18^F]FDG we have minimized bias during image analysis. *Ex vivo* studies, which included cryosectioning slices of the glioma-containing brains and autoradiography of said slices, further confirmed and supported the findings of the PET imaging studies. In addition, we assessed the expression of FRs in human glioblastoma samples obtained during patient surgery to demonstrate translational relevance suggesting that FRs may be useful for imaging and a potential treatment target for gliomas.

## 2 Materials and methods

### 2.1 Animal model and study design

The animal model was made by inoculating nine 8-month old male BDIX-ifz rats with approximately 10,000 BT4C rat glioma cells in 5 µL of growth medium injecting 2.5 mm deep into the brain after drilling a small skull hole (1 mm posterior bregma, 2 mm laterally to the right) while under 2.0-2.5% isoflurane anesthesia and 0.01 mg/kg buprenorphine and 5 mg/kg carprofen analgesia (15). Cells were grown in Dulbecco’s modified Eagle medium supplemented with 10% fetal calf serum and 0.5% penicillin-streptomycin (10,000 units/mL of penicillin and 10,000 µg/mL of streptomycin in a 10 mM citrate buffer) at 37°C in the presence of 5% CO_2_. The rats received food and water ad libitum over the course of the 32-day study, except 4 hours prior to [^18^F]FDG injection where they were fasted.

The animals were imaged with MRI on days prior to PET/CT imaging and divided into two groups which were PET/CT imaged (20-minute-static, 45 minutes post radiopharmaceutical injection) with either [^18^F]FOL or [^18^F]FDG on days 19 and 32. Two subjects underwent additional [^18^F]FOL PET/CT imaging at an earlier 16-day time point to evaluate the optimal imaging window post radiopharmaceutical injection. See **Supplementary Table 1** for image timeline distribution. During all imaging studies, animals were under light isoflurane anesthesia, and deep isoflurane anesthesia when sacrificed (via cardiac puncture followed by cervical dislocation).

All animal work was approved by the National Project Authorization Board in Finland (permission number ESAVI/6805/04.10.07/2011) and were carried out in compliance with the EU Directive 2010/EU/63 on the protection of animals used for scientific purposes.

### 2.2 Radiopharmaceutical synthesis

[^18^F]FOL (**Supplementary Figure 1**) was prepared as previously described (16). Briefly, the synthesis of [^18^F]FOL was carried out in a one-step chelation by adding 75 µL of 4 mM 1,4,7-triazacylononane-1,4,7-triacetic acid-conjugated folate to 40 µL of a 1 M sodium acetate buffer (pH 4) containing 2 mM AlCl_3_. Five GBq of [^18^F]F^-^ was then eluted to this mixture with 200 µL saline solution from a PS-HCO_3_-45 mg cartridge (ABX-Advanced Biochemical Compounds, Radeberg, Germany), and incubated at 97°C for 15 minutes. The desired product was separated from the free fluoride and other side products with semi-preparative high-performance liquid chromatography (HPLC) utilizing a Prep-Jupiter Proteo 250 × 10 mm column (Phenomenex, Torrance, CA, USA). Using 0.1% trifluoroacetic acid (TFA) in water as solvent A and 0.1% TFA in acetonitrile as solvent B on a gradient running from 8% to 18% B over 20 minutes, the desired product was eluted at 12 minutes into 25 mL of water containing 150 µL 1 M sodium bicarbonate and freshly prepared 100 µL 0.1 M gentisic acid. Our previous work (16) showed that the unlabeled precursor eluted two minutes after product collection. Acetonitrile from the collected product fraction was removed by loading the diluted product onto a Sep-Pak tC18 Plus Light 145 mg cartridge (Waters, MA, USA) and flushing with 5 mL water before being eluted with 500 µL 50% ethanol into 2 mL phosphate-buffered saline (PBS) containing 9% propylene glycol.

Quality assessment was performed *via* HPLC using a Jupiter Proteo 250 × 4.6 mm column (Phenomenex) with the same solvent combination as the semi-preparative HPLC separation running from 11% to 25% solvent B by 15 minutes with the product eluting at 9 minutes (**Supplementary Figure 2**).

### 2.3 *In vivo* imaging and analysis

Each rat received a Dotarem^®^ (Guerbet LLC, Villepinte, France) for contrast-enhanced T1 MRI (Achieva 3T MRI, Philips. Amsterdam Netherlands) with a rat-dedicated brain coil (RAPID Biomedical GmbH, Rimpar, Germany) as previously described (17). The MRI protocol included a T1-weighted MRI, then a 0.2 mL/kg injection of contrast agent, followed by a one-minute delay and a final T1-weighted image post contrast injection, which would be used to later delineate tumor boundaries. In total, 9 rats were PET/computed tomography (CT) imaged on both 19 and 32-day time points. The Inveon Multimodality scanner (Siemens Medical Solutions, Knoxville, TN, USA) was used for all PET/CT imaging and animals were imaged two-at-a-time whenever possible. Animals were injected with 39.4 ± 1.4 MBq of [^18^F]FOL or 29.6 ± 1.4 MBq of[^18^F]FDG while under anesthesia. Radiopharmaceutical dosage was selected based on previous experience imaging with [^18^F]FDG while an increased [^18^F]FOL dosage was used in an attempt to partially offset the overall lower basal brain uptake. CT was performed for attenuation correction and 20-minute PET acquisition started 45 minutes after tracer injection with data being acquired in list-mode and corrected for radionuclide decay. The collected sinograms were reconstructed with the ordered-subsets expectation maximization 3-dimensional algorithm (OSEM-3D) with 2 OSEM iterations and 18 maximum a posteriori iterations. A subset of two rats were imaged for 120 minutes starting at the time of [^18^F]FOL injection (time frames 6 × 10 s, 4 × 60 s, 5 × 300 s, 9 × 600 s).

All imaging analyses were carried out using Carimas software (http://turkupetcentre.fi/carimas/). Briefly, the contrast-enhanced MRI taken on the day prior to the PET/CT imaging, PET and CT images were aligned (manually, based on skull contour) and the tumor boundaries were established based upon the MRI. A second “expanded tumor” region was made by expanding the tumor region in all directions, which was then removed from a whole-brain region to create a “healthy brain” area with a buffer zone in between it and the MRI-established tumor volume. The PET data of the healthy brain and tumor regions were extracted and then corrected into standardized uptake value (SUV) utilizing **Supplementary Equation 1** to account for differences in injected radioactivity, leftover radioactivity in the syringe, cannula and tail, and animal weight. Tumor-to-brain uptake ratios (TBR, tumor-SUV_mean_/brain-SUV_mean_) were collected and averaged in groups by radiopharmaceutical and post tumor-grafting time point. Representative PET/CT images were made with a time-weighted mean of frames from 45 to 65 minutes with tri-cubic visual interpolation of voxels.

### 2.4 *Ex vivo* studies

After PET imaging, rats were dissected for biodistribution studies for both radiopharmaceuticals. The following organs were weighed and assayed for radioactivity using a Triathler 3**″** Nal well counter (Hidex, Turku, Finland): Whole brain (including tumor), blood, plasma, urine, muscle (flank), heart, lung, liver, pancreas, spleen, small intestine (empty), kidney, adrenal glands, salivary glands, thymus, lymph nodes, skin, and bone (femur). Results were expressed as percent of injected radioactivity dose per gram of tissue (%ID/g).

Dissected brains were immediately frozen in dry ice-cooled isopentane and then sectioned with a cryomicrotome (Leica, Wetzlar, Germany) into 8-µm and 20-µm sections, exposed for 3−4 hours on phosphor imaging plates (BAS-TR2025, Fujifilm, Tokyo, Japan), and then scanned with a BAS-5000 system (Fujifilm). The slides were temporarily frozen for storage at -70˚C, then thawed and stained with hematoxylin-eosin (H&E) following the standard procedure and then scanned with a Panoramic 250 Flash III slide scanner (3DHistech, Budapest, Hungary). Autoradiographs were then analyzed in Carimas software with overlain light micrographs to delineate tumor and brain areas. Results were expressed as photostimulated luminescence per square millimeter (PSL/mm^2^) and TBR.

### 2.5 *In vitro* [^18^F]FOL binding and blocking

The [^18^F]FOL binding specificity in tumors was assayed as previously described (17). Briefly, 20-μm cryosections of BT4C glioma-bearing rat brains that were previously frozen were slowly thawed on ice for 20 minutes before further thawing to room temperature for 5 minutes. Slides were then placed in room temperature PBS for 15 minutes. The PBS was then drained, another PBS solution containing [^18^F]FOL (0.04 MBq/mL) was added and the slides were incubated for 45 minutes at room temperature. For the blocking study, 100-fold molar excess of folate glucosamine was added to the [^18^F]FOL-PBS solution. After incubation of both non-blocked and blocked slides, cold 4˚C PBS was used to rinse them twice for 2 minutes each time and then dipped in cold plain water. Slides were allowed to dry on a rack and excess water absorbed gently with a paper towel before being exposed on phosphor imaging plates (BAS-TR2025, Fujifilm) and left to expose for two half-lives of ^18^F. Imaging plates were then scanned with a BAS-5000 system (Fujifilm) and analyzed with Carimas software.

### 2.6 Immunofluorescence and immunohistochemical staining

For immunofluorescence staining, rat brain cryosections were thawed on ice, fixed with 4% paraformaldehyde for 10 minutes and washed with PBS. Sections were then blocked in 10% bovine serum albumin for 1 hour, washed with PBS and incubated with primary antibody (FR-α working dilution 1:100, PA5-101588, Invitrogen, Waltham, MA, USA; CD68 working dilution 1:200, MCA341GA, Bio-Rad, Hercules, CA, USA; FR-β working dilution 1:50, biotinylated m909, a gift from Prof. Low) overnight at +4°C. After PBS washes, sections were incubated with secondary antibody (anti-rabbit AlexaFluor 597 working dilution 1:500, A11012, Invitrogen; anti-mouse AlexaFluor 488 working dilution 1:500, A11001, Invitrogen; Streptavidin DyLight 597 working dilution 1:50, SA-5649, Vector Laboratories, Burlingame, CA, USA), washed again with PBS, and incubated with 4′,6-diamidino-2-phenylindole (DAPI, working dilution 1:10 000, D9542, Sigma-Aldrich, St. Louis, MO, USA) for 10 minutes.

For immunohistochemical staining, sections were first blocked for endogenous peroxidases (Bloxall, SP-6000, Vector Laboratories) for 10 minutes, washed with PBS and blocked for proteins and endogenous avidin (5% normal goat serum, PK-6101, Vector Laboratories; avidin blocker, SP-2001, Vector Laboratories) for 20 minutes, washed with PBS and incubated with primary antibody (FR-α working dilution 1:100, PA5-101588, Invitrogen) with endogenous biotin blocker (SP-2001, Vector Laboratories) for 30 minutes at room temperature. After PBS wash, sections were incubated with anti-rabbit biotinylated secondary antibody (working dilution 1:200, PK-6101, Vector Laboratories) for 30 minutes, washed with PBS, and incubated with avidin-biotin enzyme complex (ABC reagent, PK-6101, Vector Laboratories) for 30 minutes, and then subjected to diaminobenzidine (DAB, SK-4100, Vector Laboratories) for antibody detection. A 10 minutes hematoxylin incubation followed for nuclear staining

### 2.7 Human tissue samples

Human formalin-fixed paraffin-embedded tissue samples (*n* = 3 glioblastoma; *n* = 2 normal-brain) were obtained from Auria Biobank (Turku University Hospital, Turku, Finland). All glioblastoma samples were taken during surgery and normal-brain samples at autopsy. The samples presented homologous findings within both groups. For immunohistochemistry, the sections were stained as previously described for anti-FR-β staining (17). For the FR-α and CD68 double immunofluorescence, the samples were stained with methods described as above for rat cryosections but, instead of a thawing process, went through xylene-ethanol series, a 2-hour antigen retrieval in citrate buffer (100 mM, pH 6.0) in pressure cooker, and then were washed with PBS.

### 2.8 Statistical analysis

Where noted, mean values with standard deviation are presented. The significance of differences between groups was calculated using an unpaired and paired two-tailed Student’s t-test for when the different groups or the same subjects were evaluated respectively. Groups separated based on radiopharmaceutical and time point after tumor grafting. Correlation between data points was evaluated using linear regression models. Reported *P*-values under 0.05 were considered statistically significant.

## 3 Results

### 3.1 Radiochemistry

[^18^F]FOL was synthesized from 5 GBq of [^18^F]F^-^ with an average radioactivity of 799 ± 84 MBq (*n =* 5) after 1.3 hours of synthesis time. The radiochemical purity was 97.5% ± 1.6 (**Supplementary Figure 2**) with a decay-corrected radiochemical yield of 27.6% ± 3.7.

### 3.2 *In vivo* imaging

**Figure 1:**
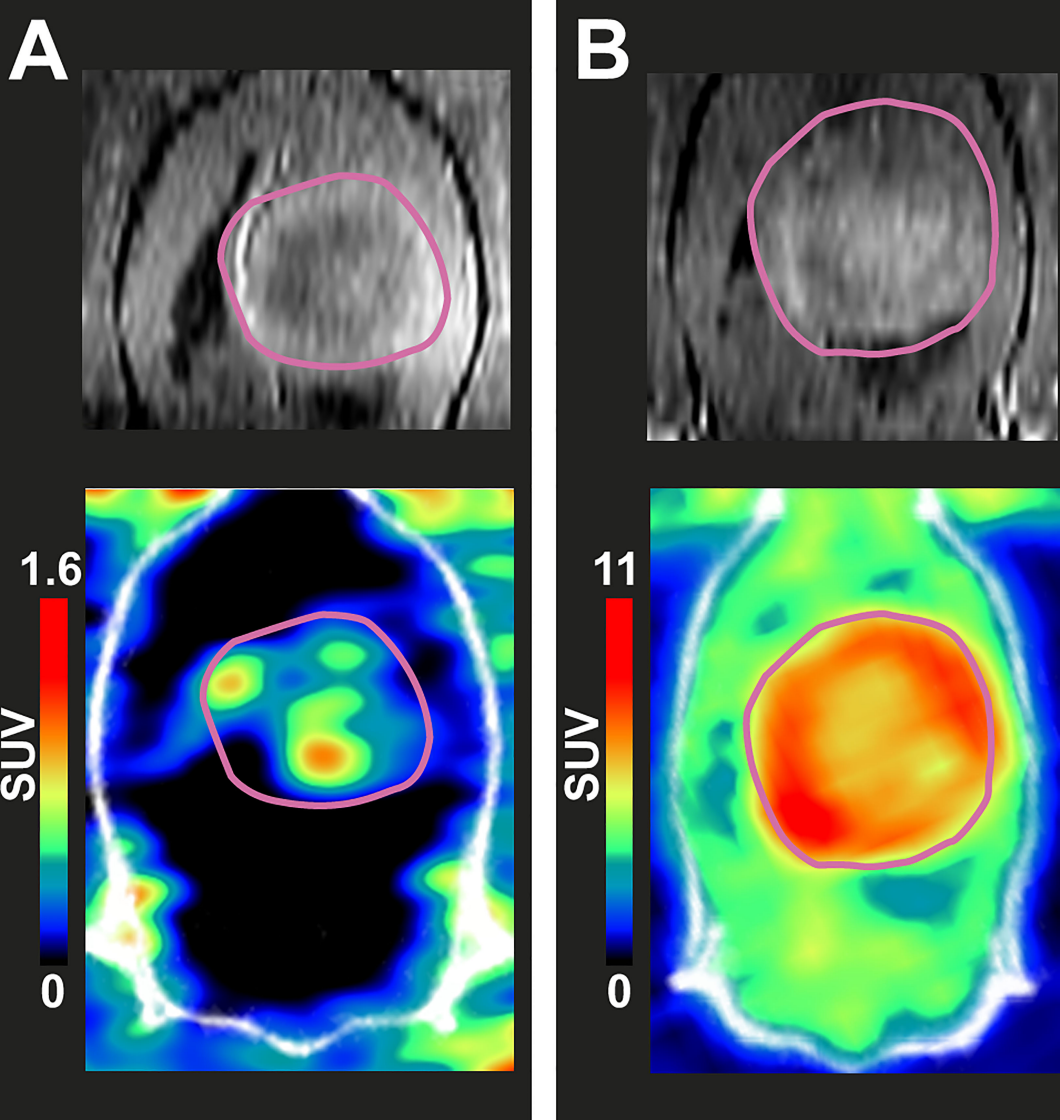
*In vivo* coronal-plane images of two BDIX rats’ brain region with BT4C glioma tumors. Top: Dotarem^®^ contrast-enhanced T1-weighted MRI at 31 days after cell inoculation. Bottom: PET/CT images of the same rats on the following day made with time-weighted mean frames from 45 to 65 minutes post-injection. Rat injected **(A)** with 40.2 MBq of [^18^F]FOL and **(B)** with 30.7 MBq of [^18^F]FDG.

**Figure 2:**
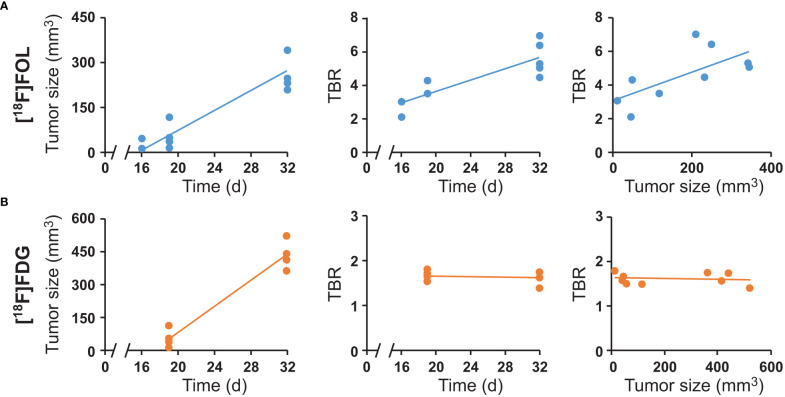
PET and contrast-enhanced MRI-derived image data of **(A)** [^18^F]FOL PET imaged subjects and **(B)** [^18^F]FDG imaged subjects. Left: tumor size measured by MRI versus time after cell inoculation. Center: Tumor-to-brain PET uptake ratios (TBR) versus time after cell inoculation. Right: TBR versus tumor size.

Tumor regions in PET images, delineated unbiasedly *via* MR images, (**Figure 1**, 32-day time point) demonstrated increased tumor uptake versus healthy brain for both [^18^F]FOL (**Figure 1A**) and [^18^F]FDG (**Figure 1B**). Increase in tumor size for all subjects through the study were clearly evident giving a 18 day time-point average size of 49.8 ± 38.1 mm^3^ (*n* = 10) and a 31 day average size of 346 ± 104 mm^3^ (*n* = 9) (**Figure 2**, left). The [^18^F]FOL TBR increased significantly (*P* = 0.0008) from (combined 16 and 19 time points, *n* = 4) 3.3 ± 1.3 to 5.7 ± 1.0 (day 32, *n* = 5, **Figure 2A**, center). [^18^F]FDG TBR values remained not significantly changed (*P* = 0.7) from 19 to 32-day time points forming a combined average of 1.6 ± 0.1 (**Figure 2B**, center, day 19 *n* = 5, day 32 *n* = 4). The difference between [^18^F]FOL and [^18^F]FDG TBR was found to be significant at both 19-day (*P* = 0.03) and 32-day (*P* < 0.001) time points. Examining the relationship between the tumor size and TBR showed an increasing trend for [^18^F]FOL (**Figure 2A**, right) and a relatively constant TBR for [^18^F]FDG (**Figure 2B**, right). Additional MRI and 32-day [^18^F]FOL images of the other 4 subjects are presented in **Supplementary Figure 3** demonstrating preferential tumor delineation from brain tissue with 16-day [^18^F]FOL time-activity curves in **Supplementary Figures 4** and **5**. Whole-body 16-day time point [^18^F]FOL PET images (**Supplementary Figure 6**) demonstrated very high kidney uptake and considerable digestive organ uptake. High [^18^F]FOL uptake was also observed outside of the skull in the scalp surgery region and uptake was also notable in the submandibular salivary glands.

### 3.3 *Ex vivo* and *in vitro* studies

[^18^F]FOL had notably low overall brain and heart uptake with high uptake occurring primarily in the kidneys and liver. The [^18^F]FDG biodistribution was in agreement with expected and literature results showing high uptake in the heart, small intestine, spleen, thymus, and brain. Detailed biodistribution results are shown in **Supplementary Figure 7**.

**Figure 3:**
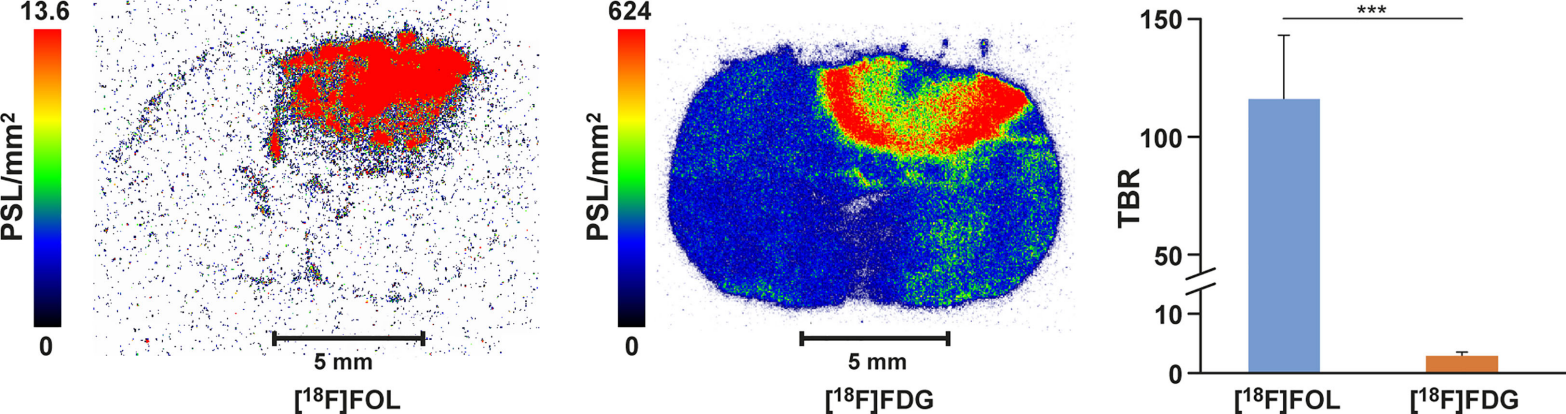
Representative autoradiographs of BDIX rat brains containing BT4C gliomas. [^18^F]FOL rat injected with 39.8 MBq, [^18^F]FDG rat injected with 26.5 MBq, and both sacrificed 70 minutes after injection. Average tumor-to-brain ratio (TBR) graphed for each radiopharmaceutical with significant difference ***, *P* < 0.0001.

Autoradiography revealed an exceptionally high [^18^F]FOL TBR of 116.1 ± 26.9 (*n =* 5 rats, *n =* 40 cryosections) while [^18^F]FDG TBR was 2.9 ± 0.6 (*n =* 3 rats, *n =* 27 cryosections) (**Figure 3**; *P* < 0.0001). Additional [^18^F]FOL autoradiographs are presented in **Supplementary Figure 8**.

**Figure 4:**
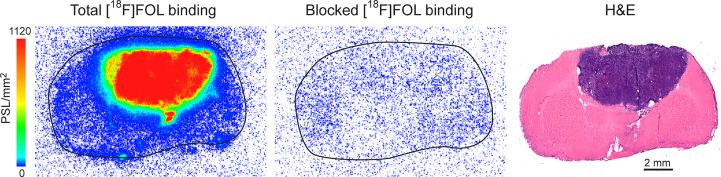
*In vitro* binding of [^18^F]FOL in BT4C glioma-bearing BDIX rat brain cryosections. Representative autoradiographs of total binding, 100-fold folate glucosamine-blocked binding, and hematoxylin-eosin (H&E) staining of adjacent sections.

*In vitro* binding studies for [^18^F]FOL (**Figure 4**) demonstrated an average total binding signal of 1211 ± 93 PSL/mm^2^ (*n =* 9) on the tumor area while the pre-incubation with a folate glucosamine gave an average blocked binding signal of 0.5 ± 0.1 PSL/mm^2^ (*n =* 9; *P* < 0.0001). Corresponding TBR for total binding were 229 ± 30 PSL/mm^2^ and blocked binding 1.1 ± 0.3 PSL/mm^2^ (*n =* 9; *P* < 0.0001).

### 3.4 Immunostaining

**Figure 5:**
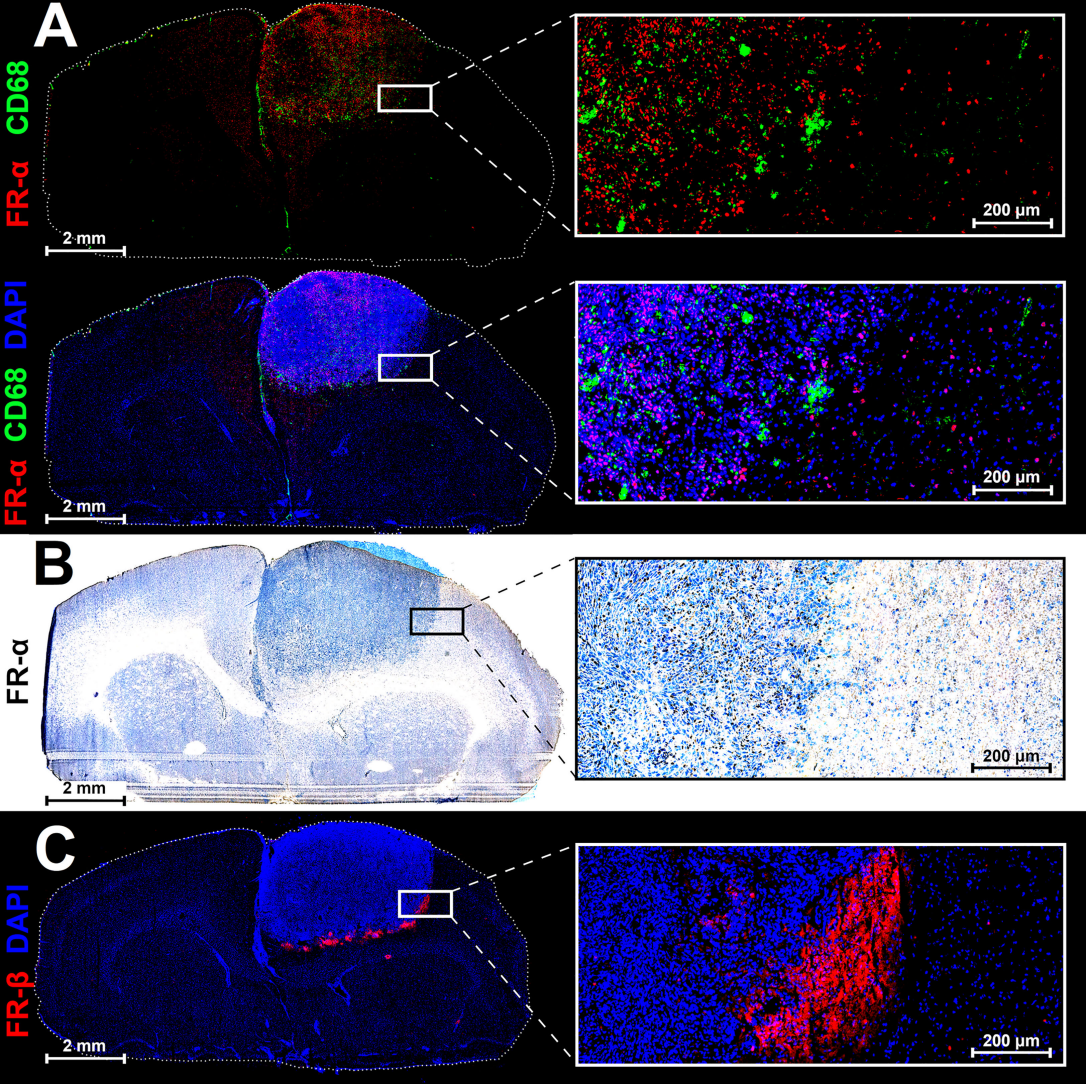
Representative immunofluorescence **(A, C)**, immunohistochemical **(B)**, and nuclei staining (DAPI) of adjacent 8-µm cryosections of a BDIX rat brain bearing a BT4C glioma. The CD68 signal is suggesting the presence of activated macrophages/microglia within the tumor while FR-β-positivity on tumor periphery is indicative of macrophage leak across the blood-brain-barrier.

For the rat brain cryosections bearing BT4C gliomas, anti-FR-α antibody was used to detect FR-α while the anti-CD68 antibody was selected to detect both activated macrophages and microglia. DAPI stain was used to detect and visualize cell nuclei and allowed for more accurate section alignment and point of reference. It was found that there was an increase in area of FR-α and CD68 fluorescence signal inside the tumor area versus adjacent healthy brain tissue (**Figure 5A**). Immunohistochemical staining for FR-α (**Figure 5B**) confirmed the positivity inside the tumor area versus healthy brain tissue. FR-β fluorescence signal was observed most notably in the tumor borderline (**Figure 5C**).

**Figure 6:**
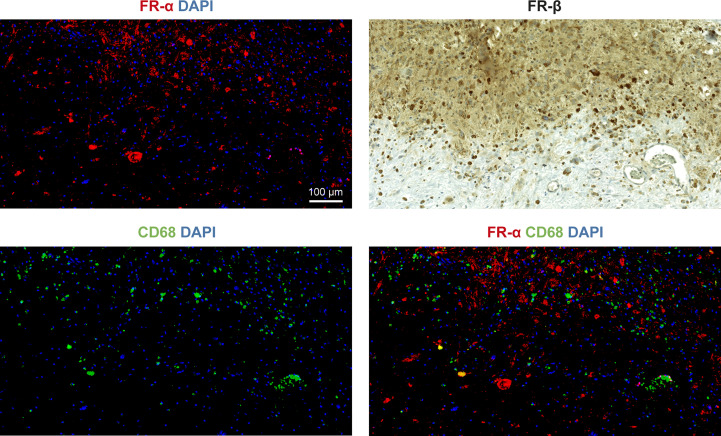
Representative immunostained human glioblastoma section region with intense FR signal. Solid tumor tissue in top of all images with less infiltrated tissue in bottom both displaying high to moderate FR-α expression (red) and high CD68 (green) with low co-localization (bottom-right). Strong FR-β expression is seen in tumor and less-infiltrated border region (top-right).

Human glioblastoma paraffin sections were stained and showed an overall high expression of FR-related signals (**Figure 6**) but with a considerable degree of heterogeneity (**Supplementary Figure 9**). The fluorescence FR-α signal was present in all tumor samples and was mostly very high apart from a few tumor regions with low signal. The regions with low FR-α signal, however, were still higher than healthy brain samples, which displayed a consistently very low signal in the white matter (**Supplementary figure 10**) and no signal in the cortex. Immunohistochemical FR-β signal in tumor samples was also highly heterogeneous ranging from low to high (**Supplementary Figure 9**). Regions that were considered low were less infiltrated by tumor tissue and coincidentally also had lower FR-α fluorescence signal. Healthy brain samples had no FR-β signal (**Supplementary Figure 10**).

## 4 Discussion

In this work, we demonstrated highly preferential uptake of [^18^F]FOL in BT4C gliomas versus adjacent non-tumor brain tissue in rats. Interestingly, despite a relatively wide investigation into [^18^F]FOL and FR-targeted PET imaging by the wider scientific community (18–20), there is only one very recent publication (21) examining the efficacy of a copper-64-labeled folate-based radiopharmaceutical for imaging gliomas. Despite this lack of glioma imaging, folate-based PET imaging research includes nearly everything from colorectal to oral cancers (22, 23).

Although no animal model will perfectly replicate a human patient, the BT4C rat glioma model was used due both practical considerations and the aggressive nature, which can also often be seen in its human counterpart. The glioma grows as a large, solid and infiltrative mass, which allows for better *in vivo* visualization in comparison to a more diffusely growing glioma, which is less conclusive to images given the spatial resolution limits of a PET camera system and the small nature of the animal model. Much like aggressive human glioblastoma, the syngeneic BT4C glioma in immunocompetent rats are highly vascularized tumors with an infiltrative behavior (24). Multiple models have used the BT4C cells grafted orthotopically into rat brains to evaluate a variety of chemotherapies and gene therapies (25). In terms of general growth characteristics, it has been described as growing expansively while invading surrounding tissue causing both neovascularization and microhemorrhages (especially of the tumor periphery) (25). Despite previously well-established morphological changes to tumor and brain vasculature, the lack of histopathological confirmation of these changes in this study is a short coming.

The PET images in this study demonstrated that despite the overall low total brain uptake, [^18^F]FOL tumor uptake reached up to 8.5-fold more than healthy brain tissue. Although evident that the total transport into the brain is considerably lower than with other radiopharmaceuticals such as [^18^F]FDG (25-fold less), it was found that the average [^18^F]FOL PET TBR was over 3.5 times higher by the end of the study. We acknowledge that the transport of [^18^F]FOL across the blood-brain-barrier (BBB) to the tumors may be primarily *via* leaking, however there may exist additional transport mechanisms highly relevant to future glioma research. We are not convinced that the spatial distribution of [^18^F]FOL perfectly matches the MRI contrast-enhancement, which highlights BBB leaking specifically. Unfortunately, the spatial resolution limitation of PET imaging combined with such a small tumor and animal model makes this inconclusive.

The implications of the uptake and distribution mechanisms with respect to other folate-based therapies are unclear due to the vast differences in glioma morphology. An increase in FR-α expression is a common trait observed in most gliomas (26) and many other cancer types (5–7) and recent studies have demonstrated that an overexpression of FR-α is very often conserved from primary tumors to secondary metastases *e.g.*, in breast cancer (27). With this in mind, further folate-targeted therapies and imaging may be worth investigating as many brain-based metastases from other cancer types have also been shown to have upregulated folate receptors (28). These brain metastases are often more viable growing near the vasculature they used to migrate into the brain, causing BBB leaking when becoming a lesion as small as 0.25 mm in diameter (29), which would shorten the distance a therapeutic compound may need to travel to these types of lesions. Larger glioma masses with poorer circulation throughout the lesion may benefit, in terms of general targeting and distribution, from a compound, which can traverse the reduced folate carrier proteins bidirectionally. Although the BBB transport of [^18^F]FOL is relatively low, the uptake in tumor tissue was found to be highly preferential. Further modifications to the [^18^F]FOL structure or brain delivery methods that result in an increase in trans-BBB passing may very likely open up the possibility for many folate-based targeting strategies.

PET imaging studies done with [^18^F]FDG were largely successful and produced results consistent with previous scientific understanding and expectations. The TBR extracted was slightly lower than other comparable literature values found [*e.g.:* TBR 2.5 (30)] though the discrepancy may be due to biochemical differences in tumor cell line, animal model, animal age, and most considerably; differing approaches to delineating tumor and non-tumor brain regions. The unbiased delineation of tumors is of the utmost importance of comparative PET imaging and much dedication was undertaken to ensure this approach was adhered to following previously reported methods (31). By imaging the rats first with a contrast-enhanced MRI on the day prior to PET imaging, a very accurate map of the tumor dimensions and boundaries was first established. This type of contrast-enhanced MRI, however, occurs primarily by BBB-leaking which also provides an entry method for radiopharmaceutical transport into the brain. It is likely that the rat brain choroid plexus, which has also been shown to express FR-α (32), was included in both the tumor region (due to large tumor size) as well as healthy brain region. When analyzing *ex vivo* autoradiographs it was noted that the radioactivity detected was mostly localized to tumor tissue.

It is interesting to note that the [^18^F]FDG TBR remained consistent throughout both study time points as the tumors tripled in volume. During this same time period, the [^18^F]FOL PET-imaged group had their TBR increase by 42%. It is unclear if the phenomenon is purely model-induced or whether other underlying factors such as an increase in inflammation may have contributed to this difference. The variability in [^18^F]FOL TBR values versus that of [^18^F]FDG are also notable though no concrete reasoning has yet to be established. The TBR values in the [^18^F]FOL autoradiography also had considerable variability suggesting that it is at least not an effect solely induced by *in vivo* imaging modality. If the BBB disruption varies significantly between subjects, it is a possible source for the variability assuming that BBB leaking is the primary method of entry.

The *ex vivo* autoradiography of [^18^F]FOL-imaged rat brain cryosections demonstrated a remarkable hundred-fold increase in tumor uptake versus healthy brain tissue, which was significantly higher than [^18^F]FDG TBR values (P > 0.001). Perhaps most interestingly, the [^18^F]FOL TBR values in autoradiographs were found to be over ten-fold higher than in the PET image data revealing a huge discrepancy. While there are many factors which can influence this, *i.e.*: PET camera resolution, sensitivity, and spill-over of adjacent radioactive areas, the autoradiography represents a more direct and accurate measurement of the uptake reality. Further investigation into why the PET images do not more accurately represent the physical reality are underway. It is hoped that these ongoing studies will characterize the influence of the three-dimensional radiochemical distribution geometry of this specific model by employing additive manufacturing techniques to create a custom PET imaging phantom. Early PET image results suggest spatial geometry to be the primary factor affecting the discrepancy observed in this study (unpublished observations). High intra-subject TBR variability was observed in both [^18^F]FOL autoradiography as well as PET TBR values when compared with [^18^F]FDG. Though, if the autoradiography values are indeed the more accurate of the data points, the difference between the autoradiography and PET data far exceeds the intra-subject PET TBR variability. The intra-subject variability in autoradiography TBR may be a number of factors, though when the value exceeds 100-fold excess in tumor tissue versus that of brain tissue, minor biochemical differences between subjects may have a drastic impact.

The [^18^F]FOL *in vitro* binding assays did not reveal as large of an uptake difference between glioma and brain tissue as the *in vivo* binding. It is likely that the mechanism of uptake of the FR-α *via* endocytosis may have been prevented by the fixation of tissue yet still allow for folate-binding. The lack of [^18^F]FOL *in vivo* blocking studies are a limitation and do not prove the efficacy of the blocking agent, though previous blocking studies carried out (16) with a differing animal disease model demonstrated clear *in vivo* blocking of [^18^F]FOL *via* a pre-injection of folate-glucosamine.

Immunohistochemical and immunofluorescence staining divulged a mix of expected and interesting signal distribution (**Figure 5**). The increase in FR-α-positive cells present in the tumor tissue versus the healthy brain was expected (**Figures 5A, B**) due to the relationship between folate consumption and DNA synthesis (1). The CD68-positivity (**Figure 5A**) was also expected due to both activated microglia and macrophages expressing the beta isoform of this receptor and comprising a large portion of the mammalian body defense network. The FR-β-positivity was present on the periphery of the tumor and in the tissue immediately adjacent to the tumor (**Figure 5C**). It is possible that the bulk of the CD68-positive signal (**Figure 5A**) was mostly activated microglia while the signal in **Figure 5C** is caused by the macrophages that are able to cross the disrupted BBB on the periphery of the rapidly expanding tumor mass.

In the healthy human brain tissue sections, FR-α fluorescence signal was especially low and likely restricted to glial cells while FR-β expression was absent. In the human glioblastoma sections, FR-α expression was detected in most, but not all glioblastoma cells, which confirmed a high degree of heterogeneity. Most of the areas examined contained exceedingly high FR-α signal levels while one tumor sample contained relatively low signal. Of the regions that displayed low signal, it was consistently higher than healthy brain samples. FR-β expression was also highly variable within the tumors, ranging from areas with negative or low staining to very intense staining.

Though we acknowledge morphological and molecular glioma-heterogeneity, the human sample staining results (**Figure 6**) show that there are some human glioblastomas that highly express FR-α and FR-β in our limited sample pool investigated. Since FR-β expression is generally indicative of activated macrophage presence, specific FR-α-targeted therapies for glioblastomas may be worth investigating. For example, the recently accelerated FDA approval of mirvetuximab (33) (Clinical Trial ID: NCT04296890), a drug-conjugated antibody specific to FR-α, may still be able to cross a disrupted BBB. Further characterization of human glioma and other brain tumor samples for the presence and overexpression of FRs is needed to ultimately demonstrate whether FR-targeted imaging and therapies will be beneficial to patients. If the rat PET imaging and autoradiography are as translational as the staining suggests, the implications are clear; further investigation is warranted.

## 5 Conclusion

The use of [^18^F]FOL for PET imaging gliomas was remarkably successful in demonstrating a clear preferential uptake in tumor tissue versus healthy brain tissue in rats. The parallels between FR expression in rat glioma and some patient-derived glioblastomas, as revealed by immunostaining, suggest that folate-based positron emission tomography could be used for the detection of gliomas in human subjects.

## Data availability statement

The original contributions presented in the study are included in the article/**Supplementary Material**. Further inquiries can be directed to the corresponding author.

## Ethics statement

Animal work was carried out in compliance with the EU directive 2010/EU/63 regarding the protection of animals used for scientific purposes and projects were approved by the National Project Authorization Board in Finland (permission number ESAVI/6805/04.10.07/2011) responsible for the oversight of animal experimentation.

## Author contributions

MM, HL, JV, SK, PE, and AR contributed to the design and conception of the project. MM, HL, JV, SK, RV, PE, JT, PS, JR, HM, NC, and X-GL contributed to the data acquisition. MM, AR, SK, and MG contributed to the interpretation of the data. MM, SK, HL, RV, MG, and AR contributed to the drafting of the manuscript. MM, HL, RV, MG, JT, PS, JR, HM, NC, PL, X-GL, and AR contributed significantly to the revision of the manuscript. All authors have approved the final manuscript, agree to be personally accountable for their own contributions and ensure the integrity of all works presented.
