# Supplementary material for: High folate receptor expression in gliomas can be detected in vivo using folate-based positron emission tomography with high tumor-to-brain uptake ratio divulging potential future targeting possibilities
Source: Front Immunol. 2023 May 18;14:1145473. doi: 10.3389/fimmu.2023.1145473 (PMC10232737; doi:10.3389/fimmu.2023.1145473)
Supplement: Supplementary file 1 [file DataSheet_1.pdf]

Supplementary Material

High folate receptor expression in gliomas can be detected *in vivo* using folate-based positron emission tomography with high tumor-to-brain uptake ratio divulging potential future targeting possibilities

Maxwell W.G. Miner[1], Heidi Liljenbäck[1,2], Jenni Virta[1], Salli Kärnä[1], Riikka Viitanen[1], Petri Elo[1], Maria Gardberg[3], Jarmo Teuho[1,4,5], Piritta Saipa[1], Johan Rajander[6], Hasan Mansour A Mansour[7], Nathan A. Cleveland[7], Philip S. Low[7], Xiang-Guo Li[1,8,9], Anne Roivainen[1,2,4,9*]

[1]Turku PET Centre, University of Turku, Turku, Finland

[2]Turku Center for Disease Modeling, University of Turku, Turku, Finland

[3]Department of Pathology, Turku University Hospital and Institute of Biomedicine, University of Turku, Turku, Finland

[4]Turku PET Centre, Turku University Hospital, Turku, Finland

[5]Department of Medical Physics, Turku University Hospital, Turku Finland

[6]Accelerator Laboratory, Turku PET Centre, Åbo Akademi University, Turku, Finland

[7]Department of Chemistry, Purdue University, West Lafayette, IN, USA

[8]Department of Chemistry, University of Turku, Turku, Finland

[9]InFLAMES Research Flagship Center, University of Turku, Turku, Finland

*** Correspondence:** Anne Roivainen: anne.roivainen@utu.fi

1 Supplementary methods

1.1 Supplementary equations

The following Supplementary Equation 1 was used to normalize data for differences in subject weight and actual injected radioactivity amounts. All radioactivity amounts were decay-corrected to the time of radiopharmaceutical injection (start of PET imaging).

$$SUV = \left(\frac{A_{ROI}}{V_{ROI}}\right) / \left(\frac{A_{sf} - A_{se} - A_c - A_t}{W_{sub}}\right)$$

Supplementary Equation 1. SUV calculation method, where A = radioactivity in kBq, V = volume in mL, and W = weight in g. Subscripts denote ROI = region of interest, sf = syringe full, se = syringe empty (after injection), c = cannula (after injection), t = subject's tail (at the end of study).

1.2 Subject grouping

The study was carried out with two separate groups of subjects imaged with either [^{18}F]FOL or [^{18}F]FDG apart from a 2-subject subset in the [^{18}F]FDG group which were dynamically PET imaged for 120-minutes with [^{18}F]FOL on day 16 to establish an optimal imaging window post radiopharmaceutical injection for future upcoming [^{18}F]FOL studies.

Supplementary Table 1 Subject characteristics and analysis quantities

	[^{18}F]FOL	[^{18}F]FDG
Subjects (*n*)	5	5*
Subject sex (F/M)	5/0	5/0
Day 15 gadolinium-enhanced MRI (*n*)	2	0
Day 16 *in vivo* PET/CT (*n*)	2†	0
Day 18 gadolinium-enhanced MRI (*n*)	5	5*
Day 19 *in vivo* PET/CT (*n*)	2‡	5*
Day 31 gadolinium-enhanced MRI (*n*)	5	4
Day 32 *in vivo* PET/CT (*n*)	5	4
Day 32 *ex vivo* biodistribution (*n*)	5	4
Day 32 *ex vivo* brain cryosection autoradiography (*n*)	5	3

* Originally *n* = 5, but one subject was euthanized mid-study due to health decline.
† Pilot study MRI and 120-minute dynamic PET to examine optimal imaging window post-injection; other PET studies 20 min static 45-min post-injection.
‡ PET imaging failed for three subjects due to unexpected camera shut down mid acquisition and file corruption.

2 Supplementary figures

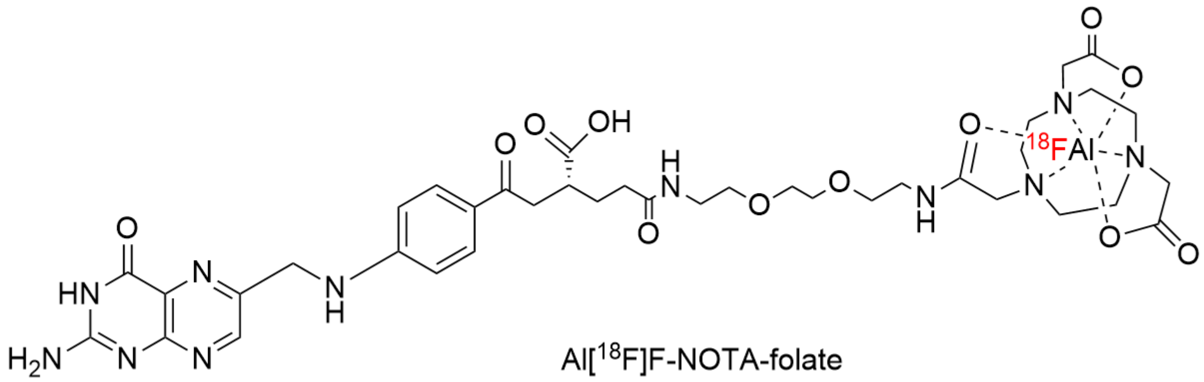

Supplementary Figure 1 Chemical structure of Al[^{18}F]F-NOTA-folate ([^{18}F]FOL).

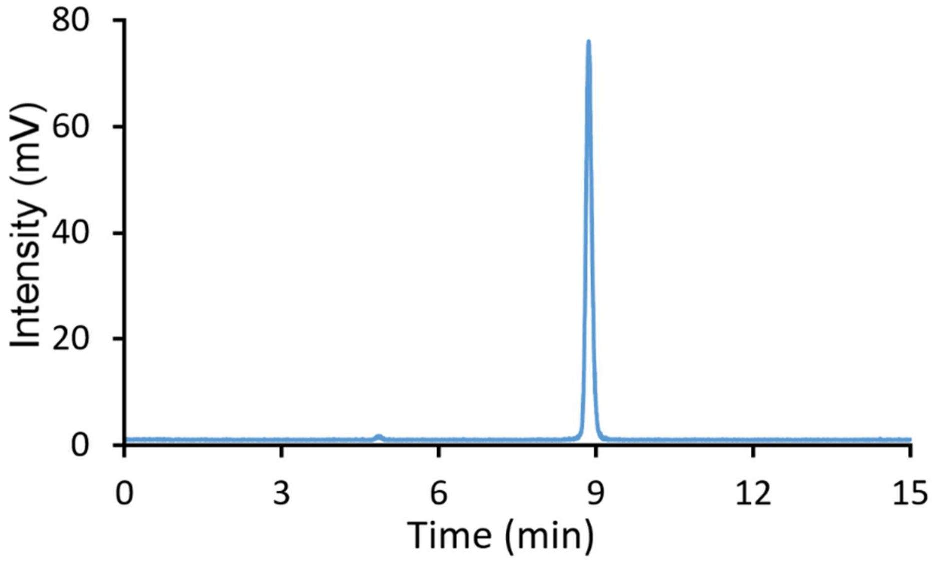

Supplementary Figure 2 Sample HPLC flow-scintillation quality control chromatogram indicating 99% [^{18}F]FOL radiochemical purity and a retention time of 8.9 minutes.

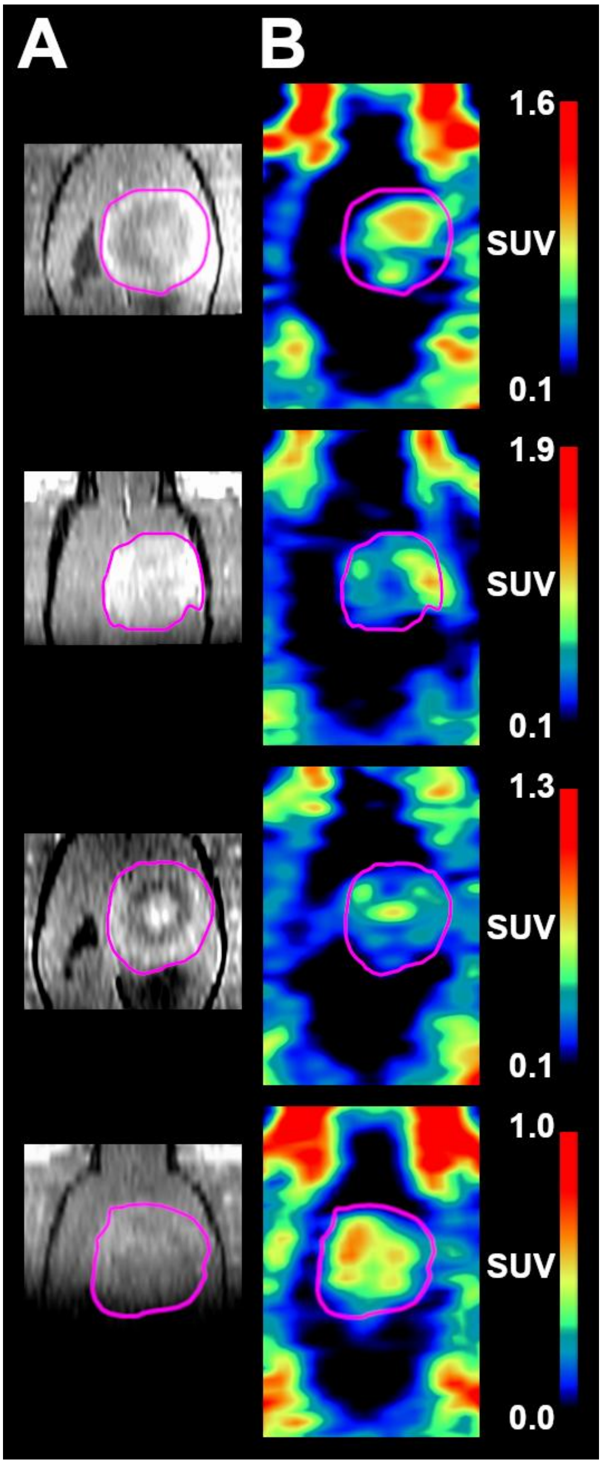

Supplementary Figure 3 Multimodal *in vivo* coronal plane image array of 32-day time point BDIX rat heads and brains bearing BT4C gliomas (outlined in magenta) with tri-cubic visual interpolation. (**A**) Gadolinium contrast-enhanced T1-weighted MRI images. (**B**) Static 20-minute [^{18}F]FOL PET images taken 45 minutes post radiopharmaceutical injection.

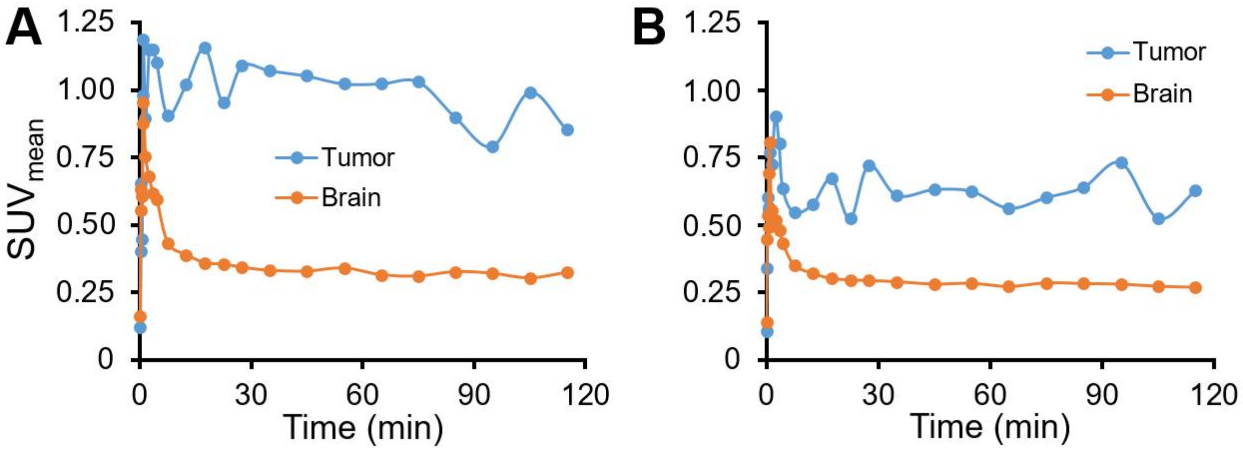

Supplementary Figure 4 Time-activity curves for two separate BDIX rat subjects 16-days post-BT4C tumor cells inoculation. Rat (**A**) was injected with 39.4 MBq of [^{18}F]FOL and rat (**B**) was injected with 40.6 MBq of [^{18}F]FOL .

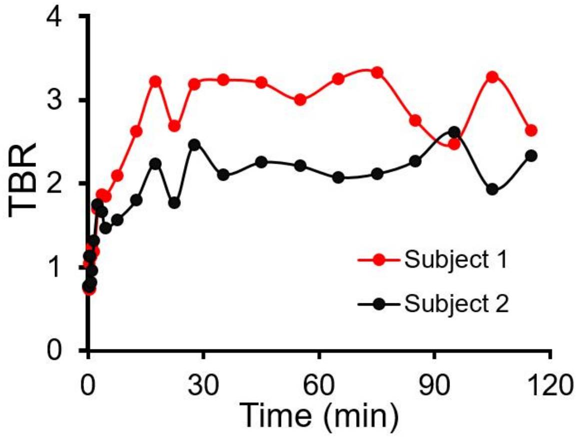

Supplementary Figure 5 Time-activity curves expressed as tumor-to-brain ratio (TBR) for the same two subjects as in Supplementary Figure 3.

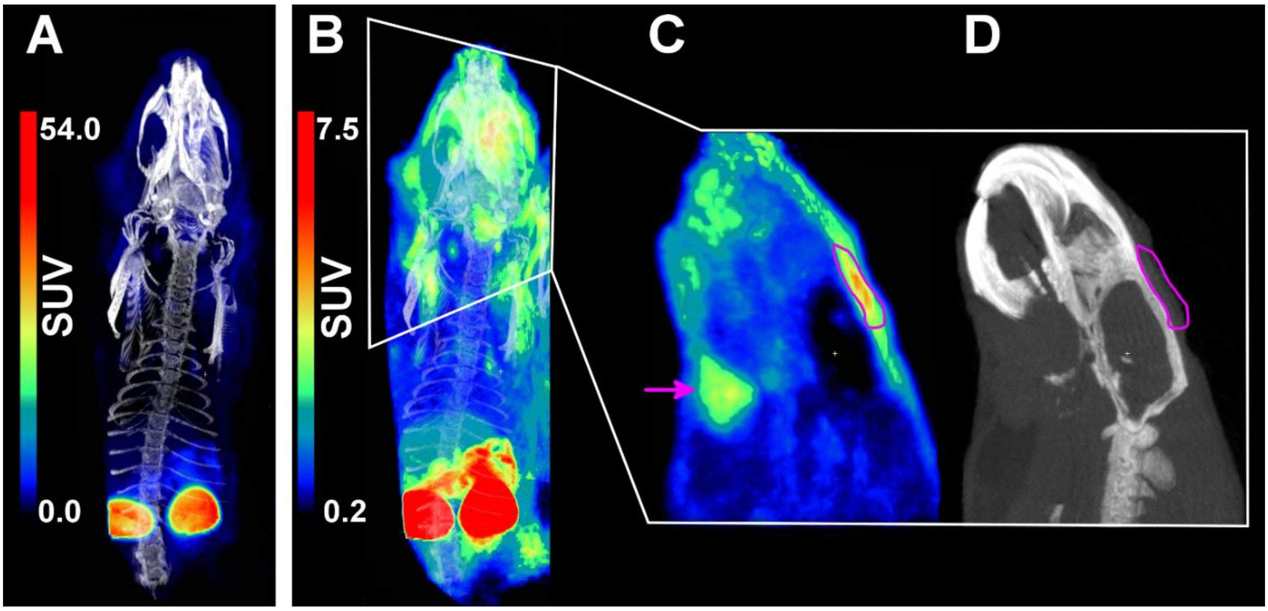

Supplementary Figure 6 Additional whole-body PET/CT, PET and CT images to demonstrate overall *in vivo* radiopharmaceutical distribution of a BDIX rat 16 days after BT4C tumor grafting injected with 40.6 MBq [^{18}F]FOL. PET images are from a time-weighted mean of frames from 5 minutes to 60 minutes with tri-cubic interpolation. (**A**) Maximum intensity projection PET/CT 3D render, whole SUV-range of image, shows very high uptake in kidneys. (**B**) Maximum intensity projection PET/CT 3D render, SUV range compressed to show high uptake in digestive organs in abdomen as well as a hot spot in the head region. (**C**) PET and (**D)** CT sagittal view cross section of head and neck region. Notable uptake in the submandibular salivary gland (magenta arrow) as well as skin surrounding the surgery site where skin was lifted to drill skull and implant tumor cells (irregular magenta oval).

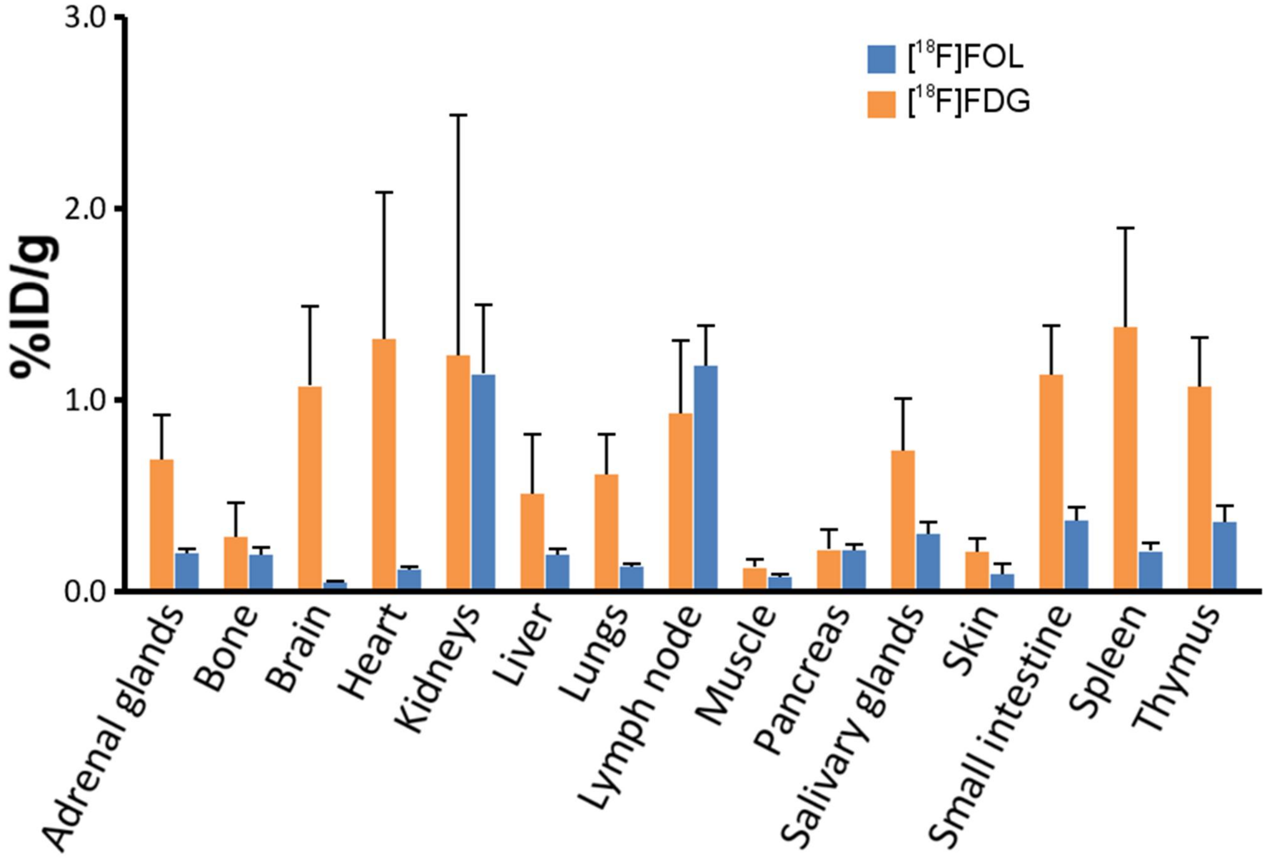

Supplementary Figure 7 *Ex vivo* biodistribution of [^{18}F]FOL (39.8 ± 0.6 MBq, *n* = 5) and [^{18}F]FDG (30.1 ± 0.3 MBq, *n* = 4) at 70-minutes post-injection in BDIX rats with brains containing BT4C gliomas grafted 32 days prior. Significant difference of overall brain uptake shown as ***, *P* < 0.0001.

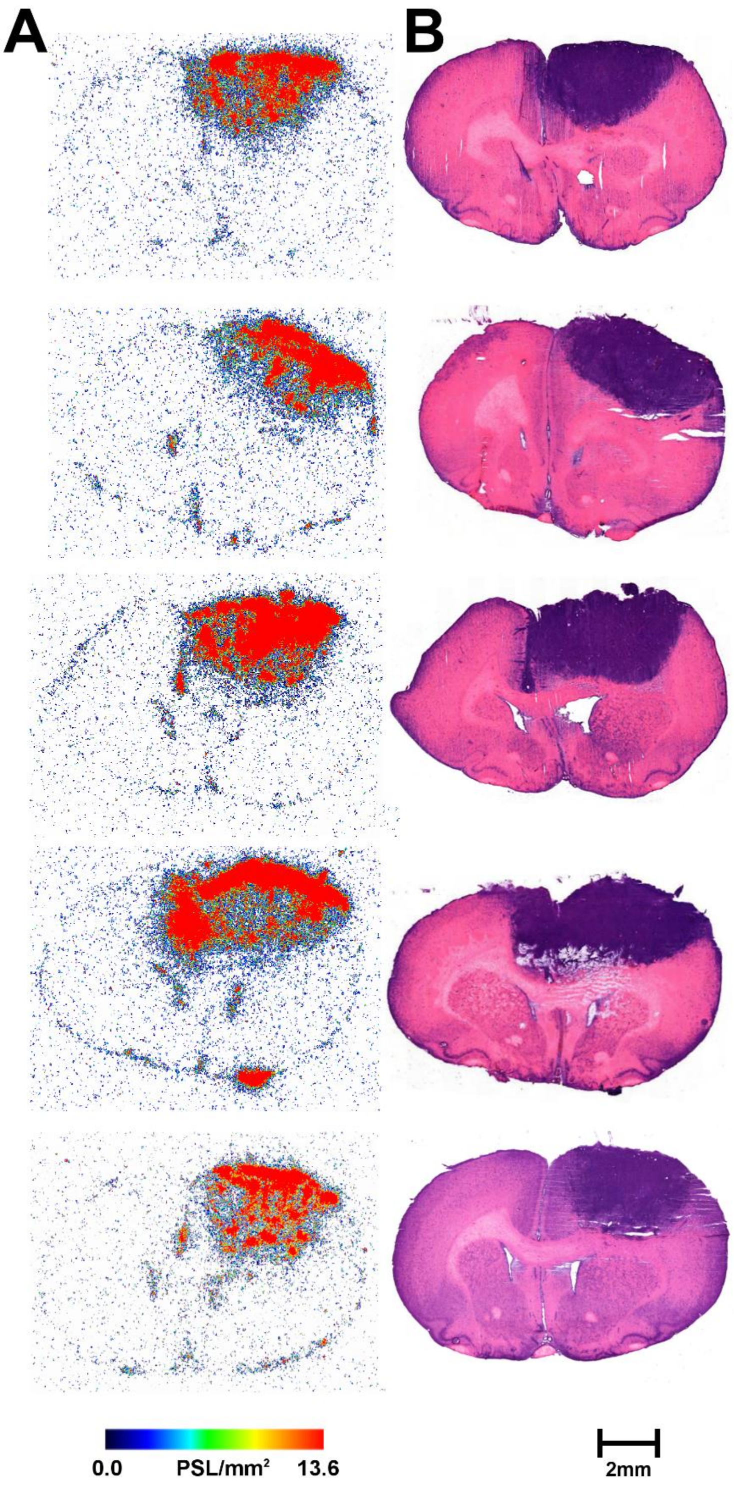

Supplementary Figure 8 Array of BT4C glioma bearing BDIX rat brains sections; (**A**) [^{18}F]FOL autoradiography and (**B**) corresponding light micrographs of H&E staining.

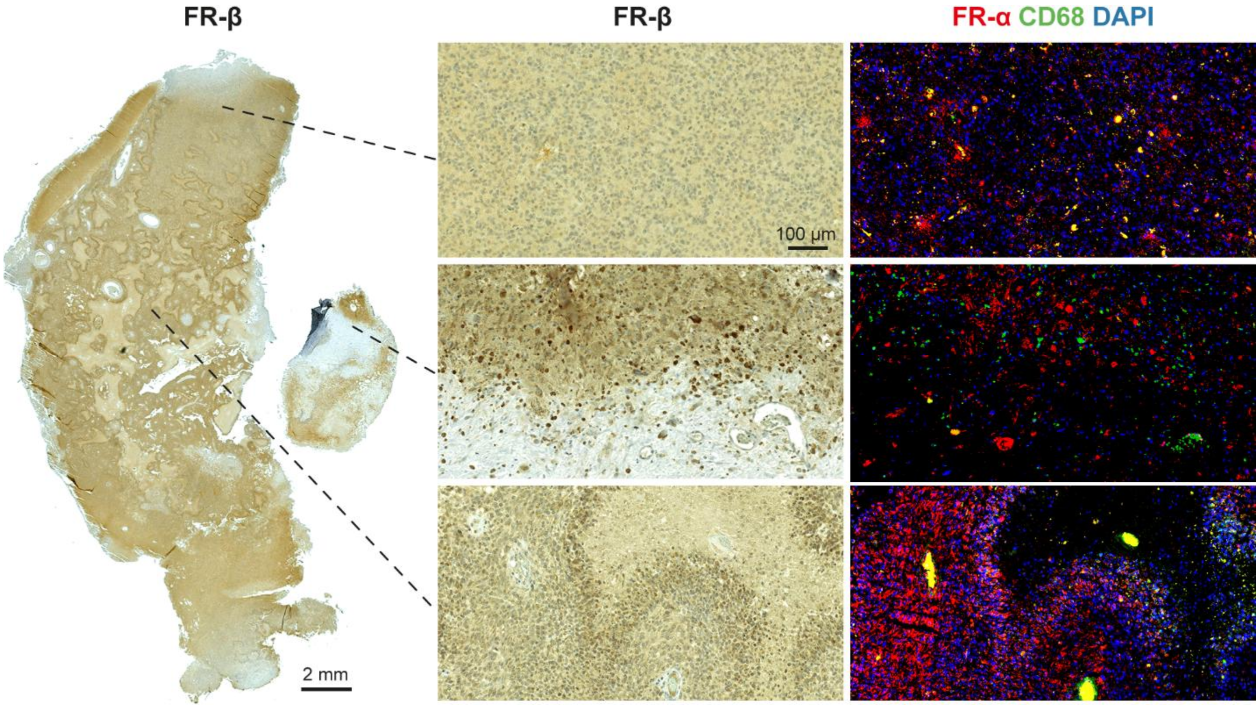

Supplementary Figure 9 Human glioblastoma formalin-fixed paraffin-embedded 6-μm section immunohistochemically stained with FR-β (left) and adjacent section of relevant zoomed in areas immunofluorescence stained (FR-α, CD68) and DAPI nuclear stain. Areas demonstrate regional tumor heterogeneity with regards to morphology and receptor expression distribution. Upper region shows tumor periphery with no FR-β, very high FR-α, moderate CD68 and low to moderate FR-α/CD68 colocalization. Middle region shows tumor border with high FR-β on the tumor side and moderate FR-β outside with very high FR-α, high CD68, and low FR-α/CD68 colocalization. Lower region shows interior necrotic zone with very high FR- β on the surrounding tissue, very high to low FR-α in surrounding tissue, and low CD68. Colocalized FR-α/CD68 spots are mostly red blood cells inside blood vessels with a CD68 positive ring in the epithelial layer.

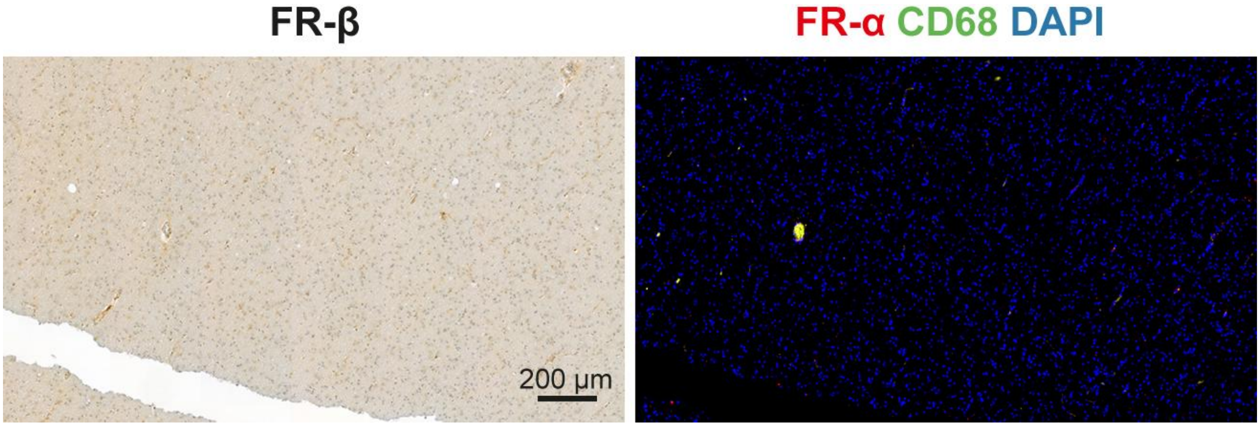

Supplementary Figure 10 Healthy human brain cortex formalin-fixed paraffin-embedded 6-µm section immunohistochemically stained with FR-β (left) and adjacent section of relevant zoomed in area immunofluorescence stained (FR-α, CD68) and DAPI nuclear stain (right). FR-α signal is present, though extremely faint, throughout cortex and in white matter the signal was absent (data not shown). CD68 signal was negative apart from blood cell staining and colocalization with FR-α in the blood vessels.
